# Supplementary material for: Comparative Pharmacokinetics and Safety of a Micellar Chrysin–Quercetin–Rutin Formulation: A Randomized Crossover Trial
Source: Antioxidants (Basel). 2025 Oct 31;14(11):1313. doi: 10.3390/antiox14111313 (PMC12649378; doi:10.3390/antiox14111313)
Supplement: Supplementary file 1 [file antioxidants-14-01313-s001.zip › Supplementary Material S1 Formulation Characterization & Stability.pdf]

Supplementary Material S1: Formulation Characterization & Stability

The purpose of this supplementary file is to provide physicochemical context that supports and helps interpret the clinical pharmacokinetic and permeability findings reported in the main manuscript. We report (i) apparent solubilized concentrations of chrysin, quercetin, and rutin from each study formulation—micellar chrysin–quercetin–rutin (LMC), non-micellar multi-flavonoid (NMC), and unformulated chrysin (UFC)—in purified water, simulated gastric fluid (SGF), and simulated intestinal fluid (SIF) using validated UHPLC quantitation; and (ii) dispersion behavior assessed by laser-diffraction particle size analysis in water (D10, D50, D90, D[4,3], Span). These characterization data are intended to enhance reproducibility (by detailing media, methods, and calibration) and to contextualize observed differences in exposure (C_{max}, AUC) and Caco-2 permeability by showing how each formulation behaves in biorelevant media. The work presented here is not a full pre-formulation/optimization campaign and is not designed to ascribe mechanism; rather, it offers standardized, comparable benchmarks of solubilization and dispersion that align with the clinical outcomes.

A. Materials and Methods

A1. UHPLC Quantification

Analyses were performed on an UltiMate 3000RS UHPLC with a quaternary pump (Thermo Fisher Scientific, MA, USA), using an Agilent Poroshell EC-18, 100 × 2.1 mm, 2.7 µm column (Agilent Technologies, CA, USA) held at 40 °C and a 0.400 mL min^{-1} flow rate; injection volume was 1.0 µL. Detection used a diode-array detector with channels at 268 nm (chrysin), 375 nm (quercetin), and 258 nm (rutin).

Mobile phases

Eluent A was 0.2% (v/v) phosphoric acid in HPLC-grade water (Fisher Scientific, ON, Canada); Eluent B was HPLC-grade acetonitrile. Mobile phases were freshly prepared and pH-checked at room temperature prior to use. After equilibrating the column at 90% A / 10% B, the sample was injected and the gradient held at 10% B for 0.30 min. Next, it is ramped linearly to 40% B at 5.00 min, then to 90% organic at 5.90 min, and returned to 10% organic by 6.00 min, followed by 3.30 min pre-injection re-equilibration.

Chemical standards

Chrysin (Millipore-Sigma, ON, Canada) was prepared as a primary stock by dissolving 10–20 mg in DMSO (Millipore-Sigma) to 10.0 mL with sonication and then diluted 1:10 with methanol for working standards. Quercetin dihydrate (Millipore-Sigma) and Rutin

trihydrate (Millipore-Sigma) were prepared as primary standards in methanol and reported on anhydrous basis for all calculations.

System Operation and Suitability

Prior to analysis, the DAD lamp was warmed for ~30 min; the pump flow was ramped gradually to 0.400 mL min^{-1} to stabilize back-pressure, and sequences were queued after line purges. External calibration was used for each analyte; linearity of $R^2 \geq 0.995$ was required. Analytes were matched for retention time to within ±0.10 min of the calibrators. Suitability parameters include having theoretical plates ≥ 5,000 and tailing factor ≤ 1.5, %RSD of five replicate injections of the mid-range standard ≤ 2.0% for peak area and ≤ 0.5% for RT, S/N at LLOQ ≥ 10.

Sample-handling note

To avoid underestimating the apparent solubilized fraction by excluding colloidal/micellar assemblies larger than common filter pores, no membrane filtration was performed on the media samples prior to UHPLC injection.

A2. Measurement of Apparent Solubility

Media

Purified water (HPLC grade), simulated gastric fluid (SGF), and simulated intestinal fluid (SIF) were prepared according to the United States Pharmacopeia (USP) compendial methods. Media pH was verified at 25 °C prior to use.

Sample Preparation

For each formulation, one capsule was placed in 10 mL of test medium (see Table S1-1 for per-portion nominal chrysin, quercetin, and rutin content).

After vortex mixing (10–15 s), tubes were sonicated at 37 °C for 60 min to account for the higher viscosity/surfactant content of biorelevant media and to promote equilibration with mixed micelles. Following sonication, tubes were briefly tapped and allowed to stand 5 min at 37 °C to let coarse bubbles dissipate.

Without further manipulation, supernatants (bulk dispersion above any visible compacted sediment) were immediately transferred to 1.5 mL glass autosampler vials with PTFE/silicone caps. No membrane filtration was performed prior to UHPLC analysis to avoid excluding supramolecular assemblies (e.g., micelles/colloids) larger than typical filter pore sizes, which would artifactually depress the measured apparent solubilized

concentration. Accordingly, the reported values represent the dissolved plus colloidally dispersed fraction that passes through the UHPLC flow path.

A3. Particle size Distribution by Laser Diffraction

Particle size distributions of the aqueous dispersions were measured by laser diffraction using a Mastersizer 3000 particle size analyzer equipped with the Hydro SM wet-dispersion unit (Malvern Panalytical, QC, Canada). Each formulation was first pre-mixed with purified water, then ~1 mL of the mixture was introduced directly into the Hydro SM accessory containing ~200 mL of water as the dispersant. The suspension was circulated through the optical measurement cell and gently stirred to maintain a homogeneous dispersion. The sample concentration was adjusted to achieve an obscuration target of ~10%. Once the target obscuration was reached and the baseline stabilized, data were acquired for 1 min while the sample continuously circulated through the cell.

Size distributions (volume-based) were calculated from the diffraction patterns using the Mastersizer software (Malvern Panalytical, QC, Canada), and are reported as D10, D50, D90, the volume-weighted mean diameter (D[4,3]), and Span=(D90–D10)/D50. Where applicable, the hydrodynamic volumes of micellar/colloidal structures were derived from the instrument's diffraction analysis outputs. Measurements were performed in triplicate (n = 3) per formulation, at ambient laboratory temperature. Between samples, the system was flushed and the background re-checked to confirm a stable baseline.

B. Results

These characterization data are provided to contextualize the clinical findings; interpretation of how solubilization and dispersion behavior relate to the observed differences in C_{max}, AUC, and Caco-2 permeability is presented in the main manuscript Discussion.

B1. Apparent Solubilities

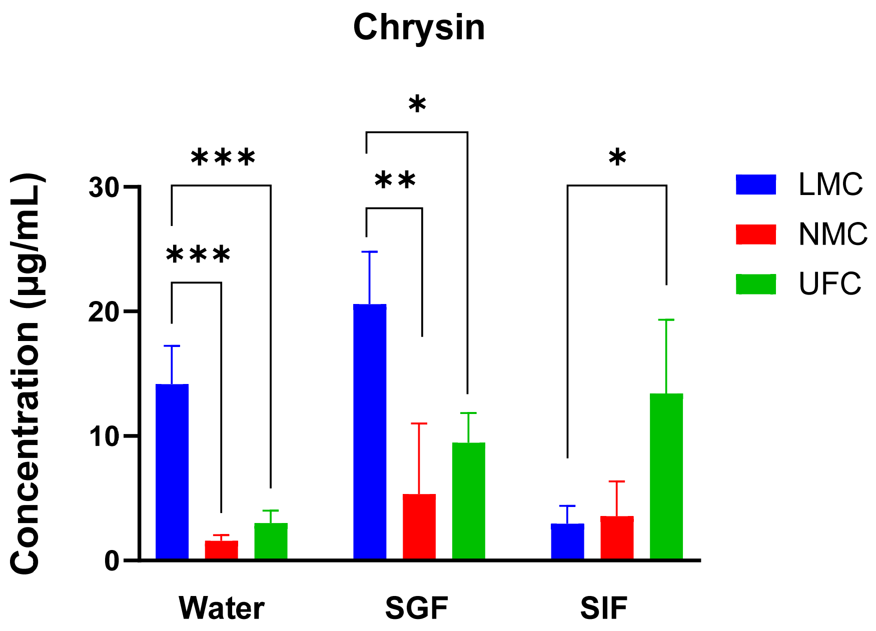

Figure S1-1. Apparent solubilized chrysin (μg·mL⁻¹) released from LMC, NMC, and UFC in water (37 °C), simulated gastric fluid (SGF, 37 °C), and simulated intestinal fluid (SIF, 37 °C). Symbols represent mean ± SD (n = 3 independent preparations per condition). Samples were vortexed and sonicated under identical conditions (see Methods) and analyzed by UHPLC without membrane filtration to capture dissolved + colloidally dispersed fractions.

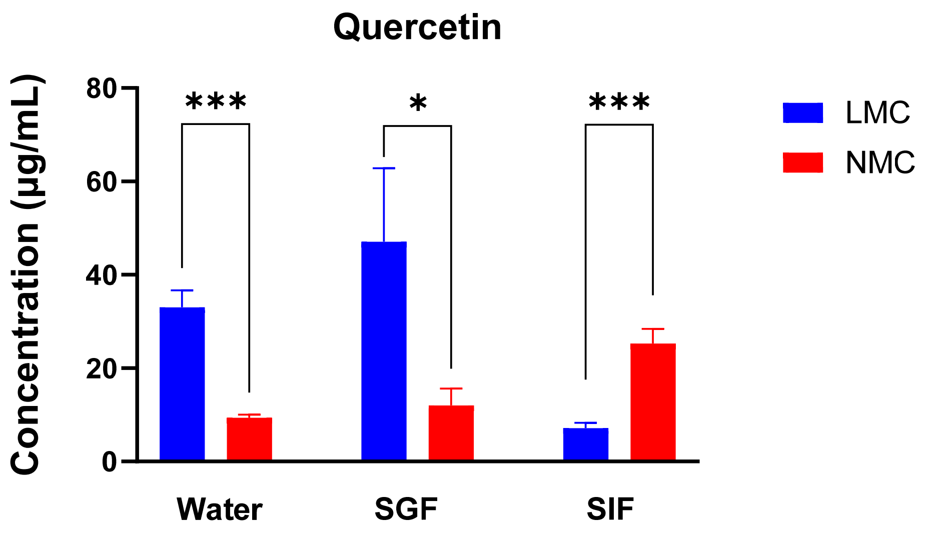

Figure S1-2. As in Figure S2-1, for quercetin (reported as anhydrous equivalents). Mean ± SD (n = 3). Note that quercetin levels in SIF can reflect pH-dependent solubilization and association with mixed micelles in SIF; values represent assay-passable material and not thermodynamic solubility.

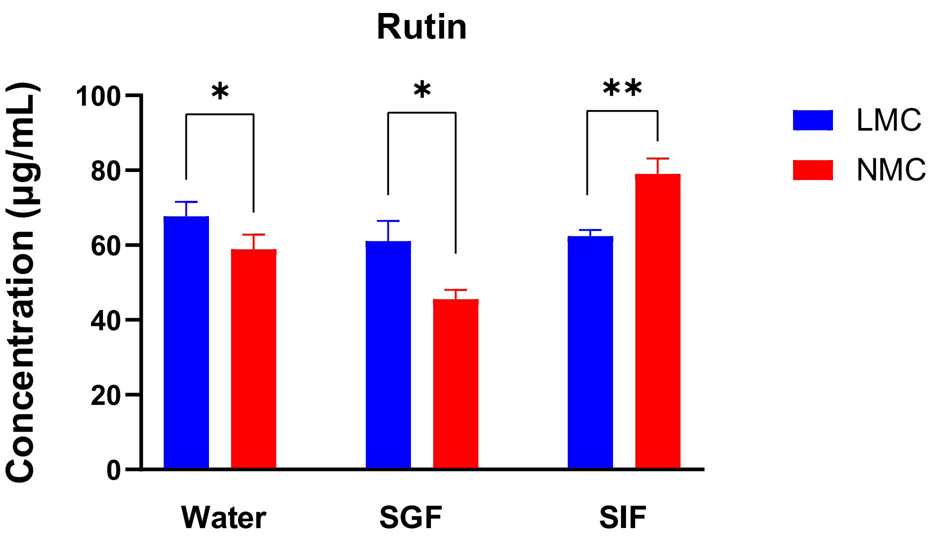

Figure S1-3. As in Figure S2-1, for rutin. Mean ± SD (n = 3). Rutin's glycoside character may yield medium-dependent behavior distinct from chrysin and quercetin; interpretation should consider the differing polarity and micelle association tendencies.

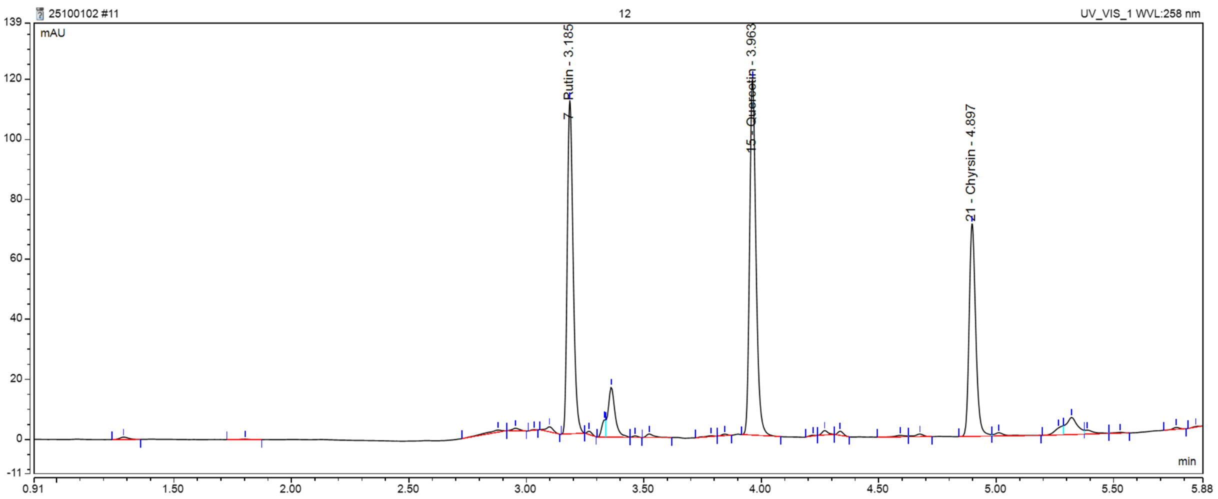

Figure S1-4. Representative UHPLC chromatogram of LMC in water. See section A for method details. Rutin, Quercetin, and chrysin display baseline separation at 258 nm. Actual quantitation was performed on 3 separate channels: 268 nm (chrysin), 375 nm (quercetin), and 258 nm (rutin).

B2. Particle Size Distributions

Table S1-1. Particle size distribution by laser diffraction (water, Hydro SM). Volume-based size metrics for aqueous dispersions measured by Mastersizer 3000 with Hydro SM (water dispersant),

reported as Dv(10), Dv(50), Dv(90), D[4,3], D[3,2], and Span = (Dv90–Dv10)/Dv50. Values are the means of triplicates unless otherwise stated; instrument obscuration was targeted at ~10% and acquisition time was 1 min at ambient temperature.

Formulation	Dv(10) (µm)	Dv(50) (µm)	Dv(90) (µm)	D[4,3] (µm)	D[3,2] (µm)	Span
LMC	12.6	84.6	206	96.9	33.2	2.291
NMC	500	603	734	610	598	0.387
UFC	9.20	25.3	43.9	26.1	18.2	1.375

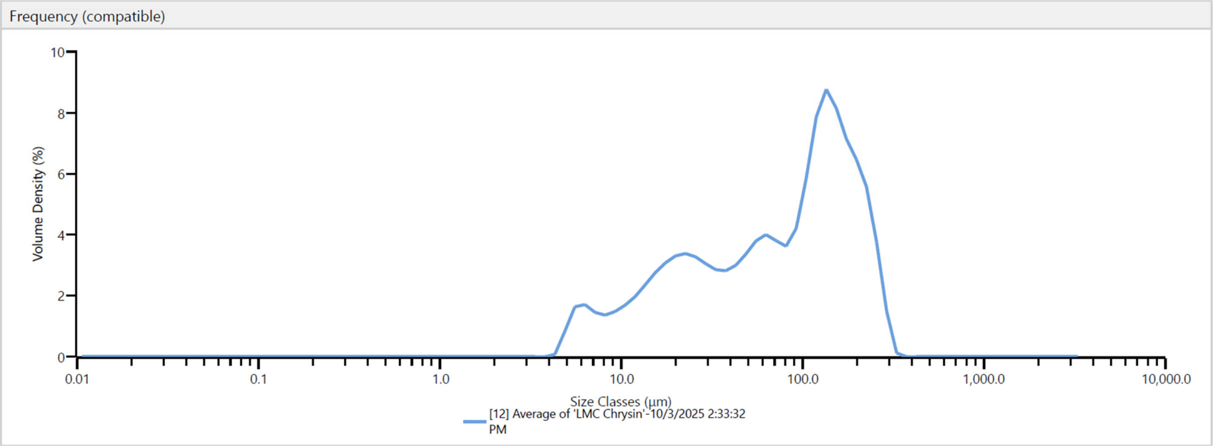

Figure S1-5. Bulk dispersion and agglomerate structure from volume-based particlesize distribution curve for LMC. Acquisition 1 min at ~10% obscuration; Mie analysis with water as dispersant. Curve shape reflects dispersion and any agglomeration under measurement conditions.

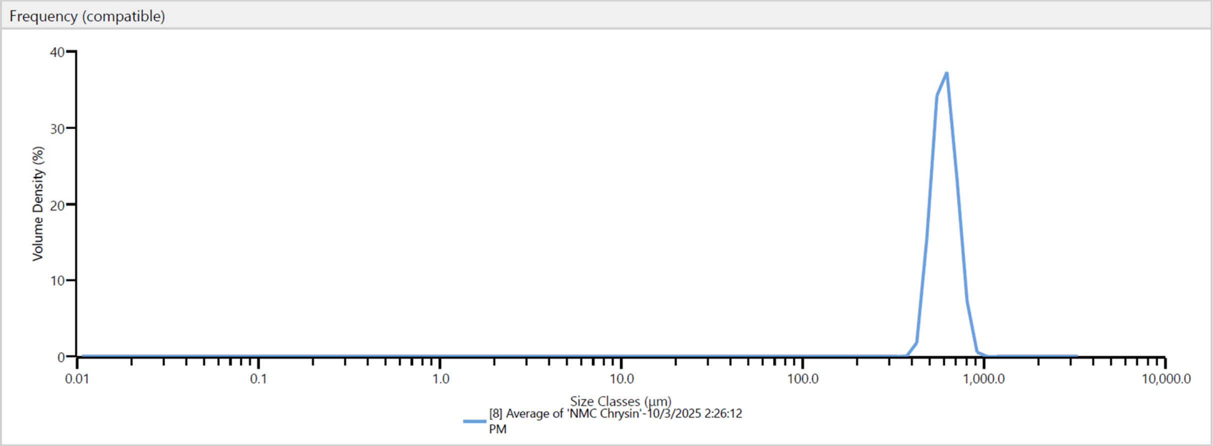

Figure S1-6. As in Figure S1-4, for NMC. The right-shifted, narrow distribution indicates larger agglomerates and lower polydispersity under these conditions, consistent with the tabled Dv values and Span.

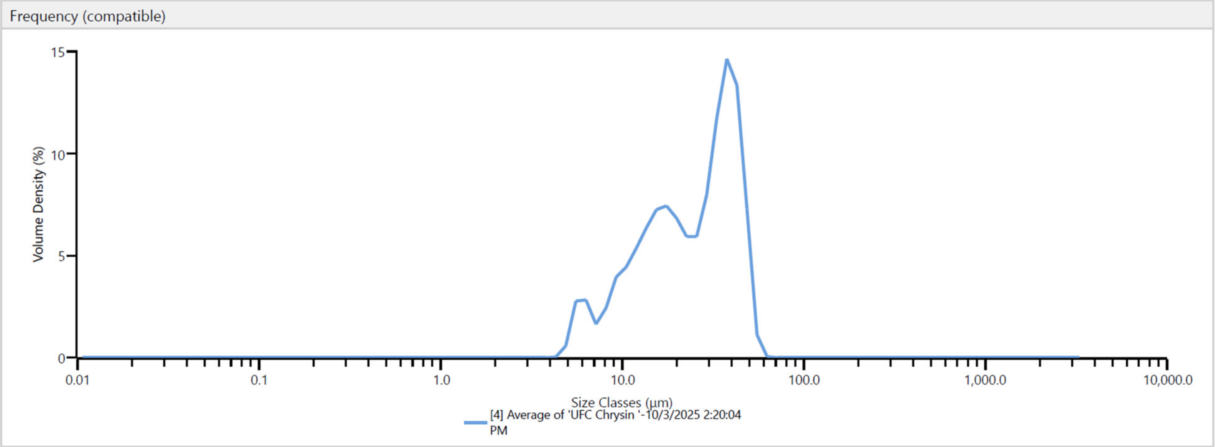

Figure S1-7. As in Figure S1-4, for UFC. The sub-100 μm mode indicates finer dispersions than NMC under identical preparation, with a moderate Span.
