# Supplementary material for: Comparative Pharmacokinetics and Safety of a Micellar Chrysin–Quercetin–Rutin Formulation: A Randomized Crossover Trial
Source: Antioxidants (Basel). 2025 Oct 31;14(11):1313. doi: 10.3390/antiox14111313 (PMC12649378; doi:10.3390/antiox14111313)
Supplement: Supplementary file 1 [file antioxidants-14-01313-s001.zip › Supplementary Material S2 Adverse Events.pdf]

Supplementary Material S2: Adverse Events

Adverse Event Monitoring Questionnaire

To assess the safety and tolerability of the study formulations, participants were asked to complete a structured adverse event (AE) questionnaire 24 hours after each treatment period. The form was designed to capture both expected and unexpected symptoms, graded by severity using a standardized 5-point scale:

Supplementary Table S2-1. Adverse Event Monitoring Criteria

Severity Score	Definition
0	No symptoms
1	Mild (noticeable but easily tolerated; no intervention needed)
2	Moderate (some interference with daily activity; no medical treatment required)
3	Severe (marked interference with daily life; may require medical intervention)
4	Life-threatening or disabling

Participants rated the severity of the following predefined symptoms:

Symptom	No symptoms	Mild	Moderate	Severe	Life-threatening
Bloating (feeling of fullness or pressure)					
Constipation					
Diarrhea					
Heartburn					
Abdominal pain/cramping/knotted sensation					
Rash					
Nausea					
Dizziness					
Blurred vision					

In addition to the severity ratings, participants responded to the following supplemental questions:

- How long did the symptoms last?
- Did the adverse events affect your daily activities or quality of life?

- Did you require any additional medication or treatment?
- Did you inform your healthcare provider about the symptoms?
- Were the symptoms tolerable, or did they significantly impact your ability to continue the study?
- Did you experience any other symptoms not listed above? Please describe.

Adverse Events Report

Completed questionnaires were reviewed by study staff, and any symptom rated ≥2 was flagged for follow-up. The results of the above questionnaires answered by the participants are summarized in the table below.

Supplementary Table S2-2. Adverse Events Report

Adverse Events			Week 1	Week 2	Week 3	Week 4
Total number of participants (*n*)	15					
Participants reporting AE (*n*)			1 (6%)	1 (6%)	2 (13%)	2 (13%)
Participants reporting type of AE (*n*)						
		Symptoms				
		Bloating	1 (6%)	1 (6%)	1 (6%)	1 (6%)
		Constipation	0 (0%)	0 (0%)	0 (0%)	0 (0%)
		Diarrhea	0 (0%)	0 (0%)	0 (0%)	0 (0%)
		Heartburn	1 (6%)	1 (6%)	1 (6%)	1 (6%)
		Pain or cramps	1 (6%)	1 (6%)	1 (6%)	1 (6%)
		Rash	0 (0%)	0 (0%)	1 (6%)	1 (6%)
		Nausea	0 (0%)	0 (0%)	0 (0%)	0 (0%)
		Dizziness	0 (0%)	0 (0%)	0 (0%)	0 (0%)
		Blurred vision	0 (0%)	0 (0%)	0 (0%)	0 (0%)
		Other (unrelated to treatment)	0 (0%)	0 (0%)	0 (0%)	0 (0%)

Total AE by severity (*n*)					
	Mild	3	3	3	4
	Moderate	0	0	1	0
	Severe	0	0	0	0
	Life-threatening	0	0	0	0
