# Supplementary material for: Comparative Pharmacokinetics and Safety of a Micellar Chrysin–Quercetin–Rutin Formulation: A Randomized Crossover Trial
Source: Antioxidants (Basel). 2025 Oct 31;14(11):1313. doi: 10.3390/antiox14111313 (PMC12649378; doi:10.3390/antiox14111313)
Supplement: Supplementary file 1 [file antioxidants-14-01313-s001.zip › Supplementary Material S3 Formulation Development and Optimization.pdf]

Supplementary Material S3: LMC Development and Optimization Details

Target product profile (TPP). Softgel fill pre-concentrate forming mixed micelles on dilution; maximized assay-passable chrysin in SGF/SIF at 37 °C; Caco-2 permeability improvement versus non-micellar comparators; compliant with routine QC and capsule manufacturability.

Component selection.

- **Actives:** chrysin (primary), quercetin, rutin (quantities per capsule in Table 1).
- **Carrier:** medium-chain triglycerides (MCT).
- **Primary amphiphile:** sunflower lecithin (phospholipids).
- **Ancillary excipients:** minor constituents for rheology and palatability (see Table 1); no synthetic polymers were required.

Screening workflow.

- **Vehicles tested:** MCT vs long-chain triglyceride oils.
- **Amphiphiles tested:** lecithin vs non-phospholipid emulsifiers.
- **Endpoints:** UHPLC-quantified solubilized chrysin/quercetin/rutin in **USP SGF/SIF** at 37 °C (0–240 min); visual dispersion stability; softgel fill suitability (viscosity window).
- **Down-selection criterion:** top-quartile solubilized chrysin in SIF with acceptable fill characteristics.

Process summary (CPPs).

- **Actives incorporation temperature: 30–70 °C** (kept within range to avoid degradation/crystallization on cooling).
- **Shear:** low-to-moderate until visually homogeneous; avoid aeration.
- **Cooling/hold:** controlled cooling before encapsulation; nitrogen overlay during hold.
- **Micellization mechanism:** self-assembly on aqueous dilution (no post-dilution high-shear step required).

Critical quality attributes (CQAs).

- Assay of chrysin, quercetin, rutin (90–120% target concentrations); content uniformity (fill weight variation ≤ 10%).
- Related substances within internal limits; peroxide value within specification.

- Fill mass/rheology within predefined range; capsule integrity/leak test pass.

Lot information. Clinical lot ID, manufacture date, and CoA summary are provided in **Table S3-1**. Supplementary Figure S1-1, S1-2, and S1-3 show the apparent solubilized concentration profiles for the clinical lot (water/SGF/SIF), and Supplementary Table S1-1 provides laser-diffraction size metrics from the same lot.

Table S3-1 Certificate of Analysis summary for LMC capsules

Parameter	Result
Description:	Chrysin LipoMicel
Clinical Lot:	2001082
Manufacturing Date:	November 2024
Lead (ICP-MS, ppm)	< 0.03
Mercury (ICP-MS, ppm)	< 0.02
Cadmium (ICP-MS, ppm)	< 0.02
Arsenic (ICP-MS, ppm)	< 0.2
Total Aerobic Microbial Count (cfu/g)	< 50
Total Yeast and Mold Count (cfu/g)	< 50
E. coli (in 10 g)	Absent
Salmonella (in 10 g)	Absent
S. aureus (in 10 g)	Absent
P. aeruginosa (in 10 g)	Absent
Chrysin (HPLC, %)	31.6%
Quercetin (HPLC, %)	7.11%
Rutin (HPLC, %)	7.73%
